# Poly[dimethyl­ammonium [tris­(μ_2_-formato-κ^2^ *O*:*O*′)cadmate(II)]]

**DOI:** 10.1107/S1600536810046830

**Published:** 2010-11-20

**Authors:** Shan Gao, Seik Weng Ng

**Affiliations:** a College of Chemistry and Materials Science, Heilongjiang University, Harbin 150080, People’s Republic of China; b Department of Chemistry, University of Malaya, 50603 Kuala Lumpur, Malaysia

## Abstract

In the coordination polymer, {(C_2_H_8_N)[Cd(CHO_2_)_3_]}_*n*_, the Cd^II^ atom lies on a special position of 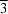 site symmetry in an octa­hedron of O atoms. The formate unit bridges the metal atoms, generating a three-dimensional polyanionic framework. The disordered cations occupy the cavities within the framework, and are N—H⋯O hydrogen-bonded to the framework.

## Related literature

For the tris­(formato)zincate cation, see; Fortier & Creber (1985 ▶); Marsh (1986 ▶). Tris(formato)cadmate is not isotypic to the aforementioned Zn structures.
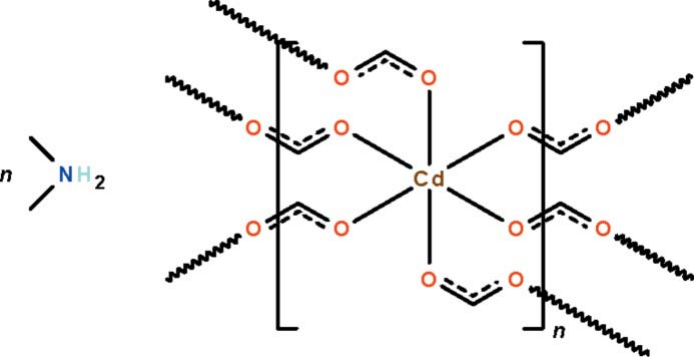


## Experimental

### 

#### Crystal data

(C_2_H_8_N)[Cd(CHO_2_)_3_]
*M* *_r_* = 293.55
Trigonal, 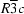

*a* = 8.5121 (4) Å
*c* = 23.0022 (9) Å
*V* = 1443.36 (9) Å^3^
*Z* = 6
Mo *K*α radiation
μ = 2.27 mm^−1^
*T* = 293 K
0.22 × 0.19 × 0.15 mm

#### Data collection

Rigaku R-AXIS RAPID diffractometer
Absorption correction: multi-scan (*ABSCOR*; Higashi, 1995 ▶) *T*_min_ = 0.635, *T*_max_ = 0.727
4250 measured reflections
370 independent reflections
352 reflections with *I* > 2σ(*I*)
*R*_int_ = 0.024

#### Refinement

*R*[*F* ^2^ > 2σ(*F* ^2^)] = 0.022
*wR*(*F* ^2^) = 0.055
*S* = 1.09
370 reflections
33 parameters
9 restraints
H-atom parameters constrained
Δρ_max_ = 0.73 e Å^−3^
Δρ_min_ = −0.36 e Å^−3^

### 

Data collection: *RAPID-AUTO* (Rigaku, 1998 ▶); cell refinement: *RAPID-AUTO*; data reduction: *CrystalStructure* (Rigaku/MSC, 2002 ▶); program(s) used to solve structure: *SHELXS97* (Sheldrick, 2008 ▶); program(s) used to refine structure: *SHELXL97* (Sheldrick, 2008 ▶); molecular graphics: *X-SEED* (Barbour, 2001 ▶); software used to prepare material for publication: *publCIF* (Westrip, 2010 ▶).

## Supplementary Material

Crystal structure: contains datablocks global, I. DOI: 10.1107/S1600536810046830/hg2747sup1.cif

Structure factors: contains datablocks I. DOI: 10.1107/S1600536810046830/hg2747Isup2.hkl

Additional supplementary materials:  crystallographic information; 3D view; checkCIF report

## Figures and Tables

**Table 1:** Hydrogen-bond geometry (Å, °)

*D*—H⋯*A*	*D*—H	H⋯*A*	*D*⋯*A*	*D*—H⋯*A*
N1—H1⋯O1	0.88	1.99	2.84 (7)	163

**Fig. 1.**
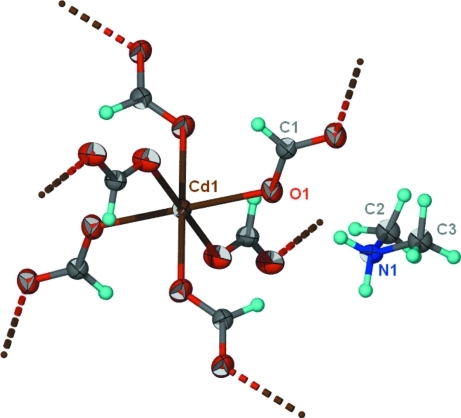
Thermal ellipsoid plot (Barbour, 2001) of a portion of poly[dimethylammonium tris(formato)cadmate] at the 50% probability level; hydrogen atoms are drawn as spheres of arbitrary radius.

## supplementary crystallographic information

### Comment

For some hydrothermal syntheses involving carboxylic acids, the *N*,*N*-dimethylformamide that is used as solvent is partially converted to the dimethylammonium cation, whose charge is balanced by the carboxylate ion. In the present study, the attempt to synthesize a coordination compound of a cadmium carboxylate yielded the tris(formato)cadmate anion (Scheme I). In the salt (Fig. 1), the cadmium atom lies on a special position of 3 site symmetry in an octahedron of O atoms. The formate unit bridges the metal atoms to generate a three-dimensional polyanionic framework, whose cavities are occupied by disordered cations.

A similar tris(formato)zincate(II) has been reported; the compound was synthesized directly from a zinc salt and formic acid in DMF medium (Fortier & Creber, 1985; Marsh, 1986). The later study has assumed the cation to be the formamidine cation, (NH_2_)CH(NH_2_)^+^. Possibly, the cation is the dimethylammonium cation.

### Experimental

*N*,*N*-Dimethylformamide (10 ml), water (1 ml), ethanol (1 ml), formic acid (0.1 ml), cadmium nitrate (5 mmol), 1,10-phenanthroline (5 mmol) and benzoic acid (5 mmol) were heated in a 23-ml Teflon-lined autoclave at 383 K for 3 days. After slow cooling the autoclave to room temperature, colorless crystals were obtained.

### Refinement

Hydrogen atoms were placed in calculated positions (C–H 0.93, N–H 0.88 Å) and were included in the refinement in the riding model approximation, with *U*(H) set to 1.2–1.5*U*_eq_(C,*N*).

The dimethylammonium cation was allowed to refine off the special position; the two N–C distances were restrained to 1.50±0.01 Å and the C···C distance to 2.35±0.01 Å. The anisotropic temperature factors of the carbon atoms were restrained to be nearly isotropic.

### Crystal data

**Table:**

(C_2_H_8_N)[Cd(CHO_2_)_3_]	*D*_x_ = 2.026 Mg m^−^^3^
*M**_r_* = 293.55	Mo *K*α radiation, λ = 0.71073 Å
Trigonal, *R*3*c*	Cell parameters from 3921 reflections
Hall symbol: -R 3 2" c	θ = 3.3–37.5°
*a* = 8.5121 (4) Å	µ = 2.27 mm^−^^1^
*c* = 23.0022 (9) Å	*T* = 293 K
*V* = 1443.36 (9) Å^3^	Prism, colorless
*Z* = 6	0.22 × 0.19 × 0.15 mm
*F*(000) = 864	

### Data collection

**Table:**

Rigaku R-AXIS RAPID diffractometer	370 independent reflections
Radiation source: fine-focus sealed tube	352 reflections with *I* > 2σ(*I*)
graphite	*R*_int_ = 0.024
Detector resolution: 10.000 pixels mm^-1^	θ_max_ = 27.5°, θ_min_ = 3.3°
ω scans	*h* = −11→11
Absorption correction: multi-scan (*ABSCOR*; Higashi, 1995)	*k* = −11→9
*T*_min_ = 0.635, *T*_max_ = 0.727	*l* = −29→29
4250 measured reflections	

### Refinement

**Table:**

Refinement on *F*^2^	Primary atom site location: structure-invariant direct methods
Least-squares matrix: full	Secondary atom site location: difference Fourier map
*R*[*F*^2^ > 2σ(*F*^2^)] = 0.022	Hydrogen site location: inferred from neighbouring sites
*wR*(*F*^2^) = 0.055	H-atom parameters constrained
*S* = 1.09	*w* = 1/[σ^2^(*F*_o_^2^) + (0.0419*P*)^2^ + 0.5666*P*] where *P* = (*F*_o_^2^ + 2*F*_c_^2^)/3
370 reflections	(Δ/σ)_max_ = 0.001
33 parameters	Δρ_max_ = 0.73 e Å^−^^3^
9 restraints	Δρ_min_ = −0.36 e Å^−^^3^

### Fractional atomic coordinates and isotropic or equivalent isotropic displacement parameters (Å^2^)

**Table:**

	*x*	*y*	*z*	*U*_iso_*/*U*_eq_	Occ. (<1)
Cd1	0.0000	0.0000	0.0000	0.02669 (17)	
O1	0.23112 (15)	0.21016 (15)	0.05612 (5)	0.0452 (3)	
C1	0.2265 (2)	0.3333	0.0833	0.0328 (4)	
H1A	0.1173	0.3333	0.0833	0.039*	
N1	0.578 (6)	0.252 (5)	0.0797 (17)	0.040 (4)	0.167
H1B	0.5788	0.1489	0.0798	0.048*	0.167
H1	0.4646	0.2277	0.0792	0.048*	0.167
C2	0.680 (5)	0.365 (4)	0.0282 (14)	0.041 (4)*	0.167
H2A	0.6061	0.3181	−0.0061	0.062*	0.167
H2B	0.7899	0.3619	0.0232	0.062*	0.167
H2C	0.7079	0.4881	0.0344	0.062*	0.167
C3	0.676 (7)	0.364 (4)	0.1320 (15)	0.041 (4)*	0.167
H3A	0.6210	0.2961	0.1667	0.062*	0.167
H3B	0.6687	0.4732	0.1322	0.062*	0.167
H3C	0.8010	0.3952	0.1306	0.062*	0.167

### Atomic displacement parameters (Å^2^)

**Table:**

	*U*^11^	*U*^22^	*U*^33^	*U*^12^	*U*^13^	*U*^23^
Cd1	0.02630 (19)	0.02630 (19)	0.0275 (2)	0.01315 (10)	0.000	0.000
O1	0.0397 (6)	0.0420 (6)	0.0554 (7)	0.0215 (5)	−0.0121 (5)	−0.0206 (5)
C1	0.0301 (8)	0.0326 (11)	0.0367 (10)	0.0163 (5)	−0.0006 (4)	−0.0011 (8)
N1	0.040 (8)	0.028 (6)	0.055 (8)	0.019 (4)	−0.006 (7)	0.002 (7)

### Geometric parameters (Å, °)

**Table:**

Cd1—O1^i^	2.2841 (10)	N1—C3	1.505 (10)
Cd1—O1	2.2841 (10)	N1—H1B	0.8800
Cd1—O1^ii^	2.2841 (10)	N1—H1	0.8800
Cd1—O1^iii^	2.2841 (10)	C2—H2A	0.9600
Cd1—O1^iv^	2.2841 (10)	C2—H2B	0.9600
Cd1—O1^v^	2.2841 (10)	C2—H2C	0.9600
O1—C1	1.2384 (14)	C3—H3A	0.9600
C1—O1^vi^	1.2383 (14)	C3—H3B	0.9600
C1—H1A	0.9300	C3—H3C	0.9600
N1—C2	1.499 (10)		
			
O1^i^—Cd1—O1	180.00 (5)	C2—N1—C3	105.3 (8)
O1^i^—Cd1—O1^ii^	91.20 (4)	C2—N1—H1B	110.7
O1—Cd1—O1^ii^	88.80 (4)	C3—N1—H1B	110.7
O1^i^—Cd1—O1^iii^	91.20 (4)	C2—N1—H1	110.7
O1—Cd1—O1^iii^	88.80 (4)	C3—N1—H1	110.7
O1^ii^—Cd1—O1^iii^	91.20 (4)	H1B—N1—H1	108.8
O1^i^—Cd1—O1^iv^	88.80 (4)	N1—C2—H2A	109.5
O1—Cd1—O1^iv^	91.20 (4)	N1—C2—H2B	109.5
O1^ii^—Cd1—O1^iv^	88.80 (4)	H2A—C2—H2B	109.5
O1^iii^—Cd1—O1^iv^	180.00 (7)	N1—C2—H2C	109.5
O1^i^—Cd1—O1^v^	88.80 (4)	H2A—C2—H2C	109.5
O1—Cd1—O1^v^	91.20 (4)	H2B—C2—H2C	109.5
O1^ii^—Cd1—O1^v^	180.00 (5)	N1—C3—H3A	109.5
O1^iii^—Cd1—O1^v^	88.80 (4)	N1—C3—H3B	109.5
O1^iv^—Cd1—O1^v^	91.20 (4)	H3A—C3—H3B	109.5
C1—O1—Cd1	124.71 (11)	N1—C3—H3C	109.5
O1^vi^—C1—O1	125.90 (19)	H3A—C3—H3C	109.5
O1^vi^—C1—H1A	117.1	H3B—C3—H3C	109.5
O1—C1—H1A	117.1		
			
O1^ii^—Cd1—O1—C1	151.34 (11)	O1^v^—Cd1—O1—C1	−28.66 (11)
O1^iii^—Cd1—O1—C1	60.11 (7)	Cd1—O1—C1—O1^vi^	−174.40 (11)
O1^iv^—Cd1—O1—C1	−119.89 (7)		

Symmetry codes: (i) −*x*, −*y*, −*z*; (ii) *y*, −*x*+*y*, −*z*; (iii) *x*−*y*, *x*, −*z*; (iv) −*x*+*y*, −*x*, *z*; (v) −*y*, *x*−*y*, *z*; (vi) *x*−*y*+1/3, −*y*+2/3, −*z*+1/6.

### Hydrogen-bond geometry (Å, °)

**Table:**

*D*—H···*A*	*D*—H	H···*A*	*D*···*A*	*D*—H···*A*
N1—H1···O1	0.88	1.99	2.84 (7)	163
